# Rural versus urban academic hospital mortality following stroke in Canada

**DOI:** 10.1371/journal.pone.0191151

**Published:** 2018-01-31

**Authors:** Richard Fleet, Sylvain Bussières, Fatoumata Korika Tounkara, Stéphane Turcotte, France Légaré, Jeff Plant, Julien Poitras, Patrick M. Archambault, Gilles Dupuis

**Affiliations:** 1 Department of Family Medicine and Emergency Medicine, Université Laval, Québec, QC, Canada; 2 Research Chair in Emergency Medicine Université Laval-CHAU Hôtel-Dieu de Lévis, Lévis, QC, Canada; 3 Population Health and Practice-Changing Research Group, CHU de Québec Research Centre, Québec, QC, Canada; 4 Department of Family Medicine and Emergency Medicine and Knowledge Transfer and Health Technology Assessment Group, CHU de Québec Research Centre and Evaluative Research Unit, Université Laval, Québec, QC, Canada; 5 Faculty of Medicine, University of British Columbia and Department of Emergency Medicine, Penticton Regional Hospital, Penticton, BC, Canada; 6 Intensive Care Division, Department of Anesthesiology, Université Laval, Quebec, QC, Canada; 7 Department of Psychology, Université du Québec à Montréal, Montréal, QC, Canada; National Yang-Ming University, TAIWAN

## Abstract

**Introduction:**

Stroke is one of the leading causes of death in Canada. While stroke care has improved dramatically over the last decade, outcomes following stroke among patients treated in rural hospitals have not yet been reported in Canada.

**Objectives:**

To describe variation in 30-day post-stroke in-hospital mortality rates between rural and urban academic hospitals in Canada. We also examined 24/7 in-hospital access to CT scanners and selected services in rural hospitals.

**Materials and methods:**

We included Canadian Institute for Health Information (CIHI) data on adjusted 30-day in-hospital mortality following stroke from 2007 to 2011 for all acute care hospitals in Canada excluding Quebec and the Territories. We categorized rural hospitals as those located in rural small towns providing 24/7 emergency physician coverage with inpatient beds. Urban hospitals were academic centres designated as Level 1 or 2 trauma centres. We computed descriptive data on local access to a CT scanner and other services and compared mean 30-day adjusted post-stroke mortality rates for rural and urban hospitals to the overall Canadian rate.

**Results:**

A total of 286 rural hospitals (3.4 million emergency department (ED) visits/year) and 24 urban hospitals (1.5 million ED visits/year) met inclusion criteria. From 2007 to 2011, 30-day in-hospital mortality rates following stroke were significantly higher in rural than in urban hospitals and higher than the Canadian average for every year except 2008 (rural average range = 18.26 to 21.04 and urban average range = 14.11 to 16.78). Only 11% of rural hospitals had a CT-scanner, 1% had MRI, 21% had in-hospital ICU, 94% had laboratory and 92% had basic x-ray facilities.

**Conclusion:**

Rural hospitals in Canada had higher 30-day in-hospital mortality rates following stroke than urban academic hospitals and the Canadian average. Rural hospitals also have very limited local access to CT scanners and ICUs. These rural/urban discrepancies are cause for concern in the context of Canada’s universal health care system.

## Introduction

While stroke care has improved dramatically over the last decade, little is known about post-stroke outcomes among patients treated in rural hospitals in Canada and whether they differ from those treated in academic urban hospitals. Roughly 20% of Canadians live rurally and more than 3 million patients are treated in rural Emergency Departments (ED) in Canada each year [1, 2]. Rural hospitals in Canada are often distant from tertiary referral centers and have limited in-house /access to services that are cardinal in the early diagnosis and management of acute stroke such as a CT scanner [1, 3–5]. Consequently, rural hospitals may not meet recent standards for acute stroke care [6, 7] potentially resulting in poorer outcomes than better staffed and equipped academic urban hospitals.

Stroke is one of the three leading causes of death in Canada [2] and represents more than $3.6 billion a year in physician services, hospital costs, lost wages, and decreased productivity costs [8]. Stroke survival partly reflects the quality of acute care received [9, 10], and stroke case-fatality rates are now used for hospital benchmarking within and between member countries of the Organisation for Economic Co-operation and Development (OECD) [11]. As Canada takes pride in the universal accessibility of its health care system, (Canada Health Act. 2011), equal access to quality stroke care should be provided to citizens wherever they reside [12]. To the best of our knowledge, no previous study has examined the rural versus urban mortality gaps on a national level in Canada.

The primary aim of this study was to describe variation in 30-day in- hospital mortality rates following stroke in rural and urban academic hospitals in Canada.

## Materials and methods

### Hospital selection

The rural hospitals selected were those located in rural small towns that provided 24/7 emergency physician coverage and inpatient beds for acute admissions. Statistics Canada defines a “rural small town” as a town or municipality outside the commuting zone of larger urban centres with populations of 10 000 or more [13]. Our full methodology for rural ED selection and data collection on hospital characteristics is described elsewhere [1, 4, 14]. We have piloted and published preliminary descriptive data on the same hospitals [1, 4, 5]. The urban hospitals were academic centres designated Level 1 or 2 trauma centres (as defined by Hameed et al.,[15]). We chose these facilities as by definition they are required to provide 24/7 in-house access to a wide range of services including CT scanners, ICUs and extensive access to speciality care and can thus constitute a potential “Gold Standard” of urban care. We identified these centres using the Canadian Institute for Health Information (CIHI) database. The CIHI, is an independent, not-for-profit organization created by the federal, provincial and territorial governments that collects and analyses information on health and health care in Canada and makes it publicly available. Stroke data has been reported since 2007. However, Quebec did not contribute to the CIHI database for the study period, due partly to differences in data collection methods for 30-day in-hospital stroke mortality rates. Data from the territories (Northwest Territories, the Yukon and Nunavut) were not included either as they did not meet the hospital selection criteria of our previous studies described above. Furthermore, we did not include urban community hospitals as the level of services and speciality care is difficult to ascertain. Moreover, several urban community hospitals are near the urban academic centers where patients are likely to be transferred to stroke centers.

### Stroke mortality data

We used the 30-day stroke in-hospital mortality data from the CIHI Health Indicators [16]. This risk-adjusted rate includes all-cause in-hospital deaths occurring within 30 days of first admission to an acute care hospital with a diagnosis of stroke [16]. This indicator is determined by the ratio of the number of stroke deaths in the hospital in a year to the expected number of stroke deaths in the same year. The expected number of stroke deaths is estimated from a logistic regression model that is fitted with age, sex, type of stroke and selected pre-admission comorbid diagnoses. This ratio is then multiplied by the Canadian average rate of stroke death to express a rate of in-hospital deaths per 100 first stroke admission episodes. As risk-adjustment modelling cannot entirely eliminate differences in patient characteristics among all hospitals in that year, the interpretation of each in-hospital rate has to be compared with the Canadian average for that year [16]. Data from every Canadian hospital was published on the CIHI website interactive tool at the time of conducting this study, unless they were considered low-volume rates (unstable stable data). Rates with denominators between 0 and 4 are considered low-volume. Details about how this is calculated are presented in S1 Table.

### Statistical analysis

Descriptive statistics such as frequency, percentage, median and interquartile range were used to characterise emergency services in rural and urban hospitals. To study the variation in emergency services in Canadian rural hospitals, we considered: 1) access to local ICUs; 2) 24/7 in-hospital access to laboratory services, basic X-rays, CT scanners and magnetic resonance imaging (MRI); and 3) number of annual visits to the ED. As 30-day in-hospital stroke mortality rates were not available for each year for every hospital, we compared characteristics of rural hospitals with CIHI data to those without for each year using chi-square or Fisher’s exact test for the presence of medical equipment and Wilcoxon signed-rank test for the number of annual emergency visits. Mean and 95% confidence interval (CI) were calculated for the 30-day in-hospital stroke mortality rate for urban and rural hospital separately. Risk-adjusted rates with confidence intervals that do not contain the Canadian average can be considered statistically different from the Canadian average. All analyses were performed with SAS 9.4 (SAS Institute, Inc., Cary, North Carolina, USA).

## Results

### Characteristics of participating hospitals

**Fig 1:**
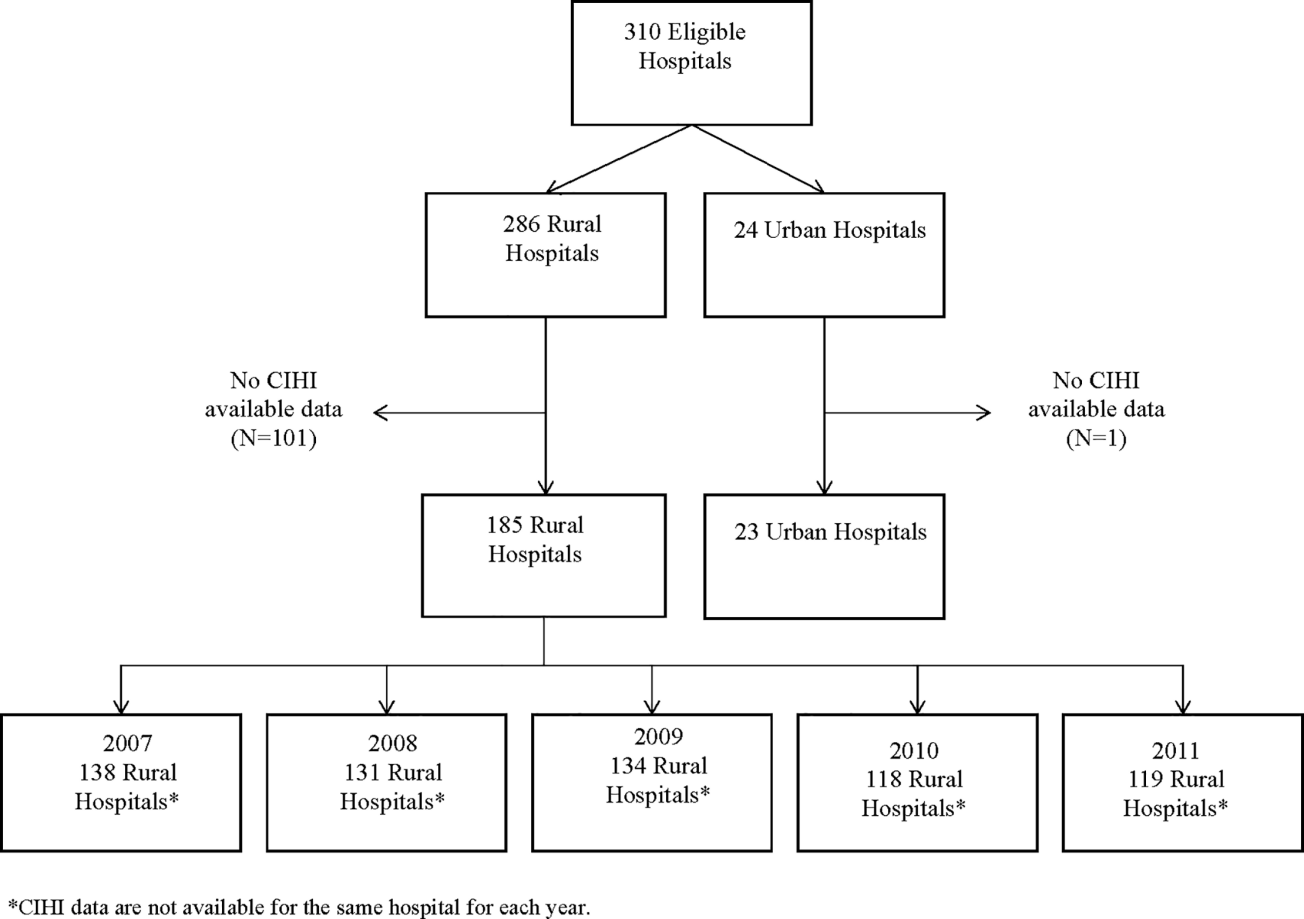
Flowchart of hospitals with CIHI available data.

Our eligibility criteria were met by 286 rural and 24 urban hospitals. Of the 24 urban hospitals, 23 (96%) provided complete data for 30-day in-hospital stroke mortality rates between 2007 and 2011. Of the 286 rural hospitals, 185 (65%) provided complete or partial information on stroke mortality rates between 2007 and 2011. Consequently, the number of rural hospitals for each year varied between 118 to 138, Fig 1. Rural hospitals received a total of 3 451 885 ED visits and urban hospitals received a total of 1 549 730 visits. In terms of services offered, every urban hospital had the five services (ICU, laboratory, radiology, CT-scanner and MRI) available 24/7. For the rural hospitals, 21% had in-hospital ICUs, 94% had laboratory, 92% had radiology, 11% had CT-scanners and 1% had MRI services.

Because of the potential for reported bias in the rural results, we compared the characteristics of rural hospitals for which CIHI data was available (rural reporting) to those without data (non-reporting). Non-reporting rural hospitals had significantly lower-volume EDs and less access to CT scans and ICU. They also had less access to basic radiology and laboratory services (Table 1).

**Table 1:** Comparison of rural hospital characteristics with and without CIHI data by year.

Characteristics	Years^5^	With CIHI Data	Without CIHI Data	P-value^6^
ICU, n (%)	2007	41 (29.9)^1^	19 (12.9)^1^	<0.01
	2008	40 (30.8)^1^	20 (13.0)^1^	<0.01
	2009	47 (35.3)^1^	13 (8.6)^1^	<0.01
	2010	43 (36.8)^1^	17 (10.2)^1^	<0.01
	2011	41 (34.8)^1^	19 (11.5)^1^	<0.01
ICU, n (%)				
	2007	133 (96.4)	137 (92.6)	0.16
	2008	128 (97.7)	142 (91.6)	0.04
	2009	130 (97.0)	140 (92.1)	0.12
	2010	113 (95.8)	157 (93.5)	0.40
	2011	114 (95.8)	156 (93.4)	0.39
Radiology 24/7, n (%)				
	2007	132 (95.7)	132 (89.2)	0.04
	2008	126 (96.2)	138 (89.0)	0.02
	2009	129 (96.3)	135 (88.8)	0.02
	2010	111 (94.1)	153 (91.1)	0.35
	2011	113 (95.0)	151 (90.4)	0.16
CT-Scan 24/7, n (%)				
	2007	25 (18.1)	6 (4.1)	<0.01
	2008	25 (19.1)	6 (3.9)	<0.01
	2009	27 (20.2)	4 (2.6)	<0.01
	2010	26 (22.0)	5 (3.0)	<0.01
	2011	27 (22.7)	4 (2.4)	<0.01
MRI 24/7, n (%)				
	2007	3 (2.2)^1^	1 (0.7)	0.35
	2008	3 (2.3)^1^	1 (0.7)	0.33
	2009	3 (2.3)^1^	1 (0.7)	0.34
	2010	3 (2.6)^1^	1 (0.6)	0.31
	2011	3 (2.5)^1^	1 (0.6)	0.31
ED annual visits, median (Q1-Q3)				
	2007	15370^2^ (9593–21176)	5475^2^ (2950–11769)	<0.01
	2008	16000^2^ (9877–21625)	5475^2^ (3013–12000)	<0.01
	2009	16000^1^ (10000–21900)	5475^3^ (3000–11769)	<0.01
	2010	17000^1^ (10558–21900)	5475^3^ (3013–12000)	<0.01
	2011	16800 (10558–21900)	6000^4^ (3120–12000)	<0.01

^1^ One missing data on characteristic data

^2^ Two missing data on characteristic data

^3^ Three missing data on characteristic data

^4^ Four missing data on characteristic data

^5^ 2007: 138 hospital with CIHI available data; 2008: 131 hospital with CIHI available data; 2009: 134 hospital with CIHI available data; 2010: 118 hospital with CIHI available data; 2011: 119 hospital with CIHI available data

^6^ Chi-square or fisher exact test for count data and Wilcoxon test for continuous data

### Thirty-day in-hospital mortality following stroke

Table 2 shows 30-day in-hospital mortality following stroke in rural and urban hospitals compared with the Canadian average. From 2007 to 2011, 30-day in-hospital mortality rates following stroke were higher in rural than in urban hospitals and higher than the Canadian average for every year except 2008 (rural average range = 18.26 to 21.04 and urban average range = 14.11 to 16.78). For all years, the Canadian average was the same as the urban hospital average.

**Table 2:** Comparison between the 30-day stroke in-hospital mortality rates for each year with the Canada average.

	Canada average	Rural average (95% IC) N	Difference^1^	Urban average (95% IC) N	Difference^1^
2007	17.7	21.04 (18.8–23.28) 138	Yes	16.78 (15.57–17.98) 23	No
2008	16.9	18.26 (16.16–20.36) 131	No	16.11 (14.5–17.7) 23	No
2009	16.0	19.71 (17.37–22.05) 134	Yes	15.86 (14.36–17.36) 23	No
2010	15.0	19.79 (17.3–22.27) 118	Yes	14.77 (13.43–16.11) 23	No
2011	14.7	19.73 (17.31–22.15) 119	Yes	14.11 (12.81–15.42) 23	No

^1^ Difference between the rural or urban average and Canada average

## Discussion

This is, to the best of our knowledge, the first study to describe variation in Canadian rural and urban academic in-hospital post-stroke mortality rates. We found that 30-day in-hospital mortality rates following stroke were higher in rural than in urban hospitals and higher than the Canadian average for every year since data collection began except for 2008. We also observed that fewer rural hospitals had in-hospital access to CT scanners, ICUs than their urban counterparts. Our findings lead us to make the following observations.

These results suggest that there may be an association between the higher stroke mortality rate in rural hospitals and their lack of stroke- pertinent emergency services. In the 2015 update of the Canadian Stroke Best Practice Recommendations, the hyper-acute stroke care guidelines recommend early identification and treatment of suspected stroke patients eligible for acute treatments (tPa or newer endovascular therapies) with “immediate” CT imaging [17]. The lack of an in-hospital CT scanner in 89% of rural hospitals would hinder physicians from timely diagnosis of or administration of acute treatments when indicated. As previously reported, at least 40% of these rural hospitals are more than 300 kms from an academic center making direct bypass to a stroke center unlikely^1^ Other variables that we did not measure, such as the presence or absence of an organized stroke team with a dedicated training program, inter-facility transfer capability, support from academic centres and even rural patient characteristics and geographic factors (e.g., distance from ED) could also have contributed to this mortality difference, as has been reported elsewhere [18].

While we cannot yet confirm the precise nature of the association between higher rural stroke mortality rates and lack of services in rural EDs, our findings are cause for concern because rural hospitals receive more than 3 million annual ED visits and stroke is a significant medical emergency. While this is the first Canadian study to report stroke data in rural hospitals, our results are congruent with those of Lichtman et al.,[19] who found rural US hospitals (Critical Access) had higher 30-day risk-standardised mortality following stroke than mainly urban (non-Critical Access) hospitals. “Critical Access” is a designation given to certain rural hospitals by the Centres for Medicare and Medicaid Services in the U.S. whose goal is to maintain essential services in rural communities [20]. A recent study comparing rural to urban citizens in Ontario, Canada’s largest province, found a similar trend of higher 30-day stroke mortality among rural than urban residents (adjusted hazard ratio 1.14; 95% confidence interval 0.99–1.32) between 2008 and March 31, 2011 [18]. The study also found that rural patients were less likely than urban patients to receive stroke unit care, brain imaging within 24 hours, carotid imaging, and consultations from neurologists, physiotherapists, occupational therapists and speech language pathologists, and were less likely to be transferred to inpatient rehabilitation facilities. Other population studies in the U.S. and China have shown that stroke mortality is not only higher in rural areas but that this mortality gap is increasing [21, 22], as advanced stroke care is improving faster in urban than in rural centres. As universal accessibility is a cardinal feature of Canada’s health care system, the stroke care discrepancies that our study has uncovered need to be better understood and rectified.

### Is the United States’ rural hospital system a model to follow?

Our study results suggest that the time has come to reduce inequities in rural health care in Canada. Looking south of the border may be a logical first step. The U.S. has passed legislation improving access to medical care and imaging services in small rural hospitals through its Critical Access Hospital (CAH) designation in 1997 [20]. An acute care general hospital can be designated a CAH by Medicare if it (1) is located in a rural area with a State Medicare Rural Hospital Flexibility Program, (2) provides 24-hour emergency care services using on-site or on-call staff members, (3) has no more than 25 inpatient beds, (4) has an average annual length of stay of 96 hours or less, and (5) is located more than 35 miles from the nearest hospital (15 miles in mountainous terrain) or is state certified as a necessary provider. Evidence suggests that CAHs have improved rural access to medical care, including to imaging services [23]. A recent report on critical access hospitals showed that 95% of CAH have a CT scanner and 83% can perform MRIs [24]. These critical care access hospitals are in reality very similar to Canada’s rural hospitals, and Canada’s discrepancy in access to care is thus particularly reprehensible given its claim to a universal-access health care system. Nevertheless, as mentioned earlier, rural versus urban stroke mortality gaps are also present in the U.S [19].

### Future interventions for improving stroke care in rural hospitals in Canada

Multiple stakeholders are currently addressing rural versus urban discrepancies in access to care. In February 2017, the College of Family Physicians of Canada, the Society or Rural Physicians of Canada in the presence of Canada’s Federal Health Minister Jane Philpott presented a strategic road map to improve access to care for rural citizens and indigenous peoples [25]. In includes the development of specific resources, infrastructure, and networks of care within local and regional health authorities to address access issues. It also proposes to develop strategies to guide implementing system-wide, coordinated, distance technology to enhance and expand local capacity, and improve access and quality health care in rural communities.

In line with this initiative, and for stroke care in particular, several interventions could contribute to reducing the rural-urban stroke mortality discrepancies. Urgently, a closer look at improving in-hospital access to CT scanners in rural hospitals that are distant from referral centers would be a first step. Improved access to CT scanners would consequently link distant rural hospitals to telestroke programs and improve upon current effective integrated stroke networks.

### Limitations/strengths

The CIHI data was available for only 65% of the hospitals we considered to be rural and for 96% of the urban academic hospitals. CIHI does not publicly report data on hospitals with fewer than five stroke cases (hospitals are identified). Further, because rural hospitals treat low volumes of stroke patients, more than 80% of reported rural rates were considered in the potentially "unstable" data range (5< = denominator<50, or expected episode<1 with numerator>0) according to CIHI's criteria. Results must thus be interpreted with caution.

A legitimate question is whether hospitals with missing data could have had lower mortality rates than those whose data are reported, thus minimizing the rural-urban stroke mortality discrepancies reported here. Further analysis suggests this is unlikely. Rural hospitals with available stroke mortality data received significantly more ED visits and were more likely to have a CT scanner and ICU than hospitals without available stroke data. Limited clinical exposure to cases in smaller centres is generally linked to poorer rather than better outcomes.

Another limitation is that data on stroke mortality was not available for the province of Quebec for the study period. According to CIHI, data collection methods and certain access restrictions to Quebec government data preclude the CIHI from publishing data on its website. This is unfortunate as Quebec is Canada‘s second most populated province (8 million). Quebec rural hospitals also have better access to CT scanners than other Canadian provinces (74% vs. less than 10%) [5, 14]. Further comparison of stroke in Quebec rural hospitals with the rural hospitals in the rest of Canada will be of interest considering their differential access to in-hospital services. CIHI data on stroke mortality in Quebec will be available in the future.

We also did not collect data on EDs in the Northwest Territories, Nunavut and Yukon as they did not uniformly meet inclusion in our initial rural ED studies. CIHI 30-day in hospital mortality following stroke data was also not uniformly available for our study period. The territories have differently managed health care systems, are more distant from tertiary centers and have a higher proportion of Indigenous peoples. Thus, potential variation in stroke mortality rates is expected. Future studies on access to rural and remote emergency services should include the territories.

Other potential limitations include the fact patient level characteristics, known to be predictive of stroke mortality, are not available in this database. CIHI data-base reports hospital-level data versus patient–level-data for this indicator. Further multivariate analyses with adjustments for other factors (not adjusted for by CIHI) such as presence of local CT/MRI/ICU/consultants would require patient-level data and will be the focus of future studies. Moreover, rural versus urban times from symptom onset and stroke treatment were not available in the CIHI database as well. Future, prospective, patient–level studies that include a measure of time from estimated symptom onset and treatment are required to further elucidate the concerning rural versus mortality gaps observed in this study.

This study has several strengths. We used an established national health outcomes database (CIHI) and presented stroke data for every year since they have been available. Rural definitions and hospital selection were achieved using rigorous criteria and levels of services were obtained directly from the hospitals themselves using brief structured interviews with hospital managers (methodology described elsewhere) [1].

## Conclusion

To the best of our knowledge, this is the first report on disparities in 30-day stroke mortality in rural vs urban Canadian academic hospitals. While the rural/urban gap in access to emergency care resources such as specialists and CT scanners is increasingly acknowledged, this is the first report on disparities in patient outcomes. This information is cause for concern in the context of Canada’s universal health care system. Future studies should address the underlying causes of such disparities.

## Supporting information

S1 Table This table describe the risk-adjusted rate of all-cause in-hospital death occurring within 30 days of first discharge from an acute care hospital with a diagnosis of stroke. It is available at: http://indicatorlibrary.cihi.ca/display/HSPIL/30-Day+Stroke+In-Hospital+Mortality.
(DOCX)
