# Supplementary material for: Rural versus urban academic hospital mortality following stroke in Canada
Source: PLoS One. 2018 Jan 31;13(1):e0191151. doi: 10.1371/journal.pone.0191151 (PMC5791969; doi:10.1371/journal.pone.0191151)
Supplement: S1 Table — It is available at: http://indicatorlibrary.cihi.ca/display/HSPIL/30-Day+Stroke+In-Hospital+Mortality. (DOCX) [file pone.0191151.s001.docx]

**S Table 1: CIHI methodology used to calculate the 30-Day Stroke in-hospital mortality**

| **Name** | **30-Day Stroke In-Hospital Mortality** |
| --- | --- |
| **Indicator Description and Calculation** |  |
| Description | The risk-adjusted rate of all-cause in-hospital death occurring within 30 days of first admission to an acute care hospital with a diagnosis of stroke  For further details, please see the [General Methodology Notes](http://indicatorlibrary.cihi.ca/download/attachments/1114124/General%20Methodology%20Notes.pdf). |
| Calculation: Description | The risk-adjusted mortality rate (RAMR) for each region was calculated by dividing the observed number of in-hospital deaths for each region by the expected number of in-hospital deaths for the region and multiplying by the Canadian average in-hospital death rate.  Unit of Analysis for Denominator Cases: Single admission |
| Calculation: Geographic Assignment | Place of residence |
| Calculation: Type of Measurement | Rate - per 100 |
| Calculation: Adjustment Applied | The following covariates are used in risk adjustment: For a detailed list of covariates used in the model, please refer to the Specification document. |
| Calculation: Method of Adjustment | Logistic regression |
| Denominator | *Description:* Total number of stroke episodes in an 11-month period *Inclusions:* 1. a. Stroke (ICD-10-CA: I60–I64; ICD-9-CM: 430–432; 433–434 with fifth digit of 1; 436) is coded as most responsible diagnosis (MRDx) but not also as a diagnosis type (2); or  b. Where another diagnosis is coded as MRDx and also a diagnosis type (2), and a diagnosis of stroke is coded as a type (1) or type (W), (X) or (Y) but not also as type (2); or  c. Where rehabilitation (ICD-10: Z50.1, Z50.4–Z50.9; ICD-9-CM: V57) is coded as MRDx and stroke as a type (1), or type (W), (X) or (Y) but not also as type (2)  2. Admission between April 1 and March 1 of the following year (period of case selection ends March 1 to allow for 30 days of follow-up)  3. Age at admission 20 years and older  4. Sex recorded as male or female  5. Admission to an acute care institution (Facility Type Code = 1)  6. Admission category recorded as urgent/emergent (Admission Category Code = U)  7. Canadian resident (Canadian postal code) *Exclusions:* 1. Records with an invalid health card number  2. Records with an invalid date of birth (non-Quebec records)  3. Records with an invalid admission date  4. Records with admission category of cadaveric donor or stillbirth (Admission Category Code = R or S)  5. Previous stroke: Records with a stroke inpatient admission [(ICD-10-CA: I60–I64; ICD-9-CM: 430–432; 433–434 with **fifth** digit of 1; 436) coded as diagnosis type (M), (1), (2), (W), (X) or (Y); Facility Type Code = 1] within one year prior to the admission date of the index episode |
| Numerator | *Description:* Number of deaths from all causes occurring in hospital within 30 days of admission for stroke *Inclusions:* 1. Discharge Disposition Code = 07 (Died)  2. Facility Type Code = 1 (Acute Care)  3. (Discharge date on death record) − (Admission date on stroke record) less than or equal to 30 days  *Exclusions:* 1. Records with an invalid discharge date |
| **Background, Interpretation and Benchmarks** | |
| Rationale | Stroke is a leading cause of death and long-term disability. Adjusted mortality rates following stroke may reflect the underlying effectiveness of treatment and quality of care. |
| Interpretation | Lower rates are desirable.  Inter-regional variations in stroke mortality rates may be due to jurisdictional and institutional differences in standards of care, as well as to other factors that are not included in the adjustment. |
| HSP Framework Dimension | Health System Outputs: Appropriate and effective |
| Areas of Need | Getting Better |
| Targets/Benchmarks | Not applicable |
| References | Hosmer DW, Lemeshow S. Confidence Interval Estimates of an Index of Quality Performance Based on Logistic Regression Models. *Stat Med* 1995(14): 2161-2172.  Mayo NE, Goldberg MS, Levy AR, et al. Changing Rates of Stroke in the Province of Quebec, Canada: 1981-1988. *Stroke* 1991;22(5): 590-595.  Mayo NE, Neville D, Kirkland S, et al. Hospitalization and Case-Fatality Rates for Stroke in Canada From 1982 Through 1991. The Canadian Collaborative Study Group of Stroke Hospitalizations. *Stroke* 1996(27): 1215-1220.  Weir N, Dennis MS. Towards a National System for Monitoring the Quality of Hospital-Based Stroke Services. *Stroke* 2001(32): 1415-1421. |
| **Availability of Data Sources and Results** |  |
| Data Sources | DAD, HMDB, MED-ÉCHO |
| Available Data Years | *Type of Year:* Fiscal *First Available Year:* 1998 *Last Available Year:* 2013 |
| Geographic Coverage | All provinces/territories |
| Reporting Level/Disaggregation | National, Province/Territory, Region, Neighbourhood Income Quintile |
| **Result Updates** | |
| Update Frequency | Every year |
| Indicator Results | *Web Tool:* Health Indicators E-publication  *URL:* <http://www.cihi.ca/hirpt/?language=en> |
| Updates | Beginning with rates based on 2003–2004 to 2005–2006 data, case selection criteria for stroke were revised to include patients transferred to rehabilitation during their index admission. In this case, stroke may not be coded as the most responsible diagnosis; these cases were previously excluded from the indicator. |
| **Quality Statement** | |
| Caveats and Limitations | Not applicable |
| Trending Issues | Beginning with 2004 rates, stroke case selection criteria were revised; therefore, comparison of rates from 2004 onward with those of previous years should be made with caution. |
| Comments | Rates are based on three years of pooled data. The reference year reflects the mid-point of a three-year period. |

This table describe the risk-adjusted rate of all-cause in-hospital death occurring within 30 days of first discharge from an acute care hospital with a diagnosis of stroke. It is available at: <http://indicatorlibrary.cihi.ca/display/HSPIL/30-Day+Stroke+In-Hospital+Mortality>
